# Effect of Cyclodextrins on the Biofilm Formation Capacity of *Pseudomonas aeruginosa* PAO1

**DOI:** 10.3390/molecules27113603

**Published:** 2022-06-03

**Authors:** Zsófia Berkl, Ildikó Fekete-Kertész, Kata Buda, Emese Vaszita, Éva Fenyvesi, Lajos Szente, Mónika Molnár

**Affiliations:** 1 Department of Applied Biotechnology and Food Science, Budapest University of Technology and Economics, 1111 Budapest, Hungary; berkl.zsofia@vbk.bme.hu (Z.B.); fekete.kertesz.ildiko@vbk.bme.hu (I.F.-K.); kata.buda.bk@gmail.com (K.B.); vaszita.emese@vbk.bme.hu (E.V.); 2 CycloLab Cyclodextrin R & D Laboratory Ltd., 1097 Budapest, Hungary; fenyvesi.e@cyclolab.hu (É.F.); szente@cyclolab.hu (L.S.)

**Keywords:** *Pseudomonas aeruginosa*, biofilm, cyclodextrins, quorum sensing, quorum quenching

## Abstract

Quorum sensing (QS) is a population-density-dependent communication process of microorganisms to coordinate their activities by producing and detecting low-molecular-weight signal molecules. In pathogenic bacteria, the property controlled by QS is often related to infectivity, e.g., biofilm formation. Molecular encapsulation of the QS signals is an innovative method to prevent the signals binding to the receptors and to attenuate QS. Cyclodextrins (CDs) may form an inclusion complex with the signals, thus reducing the communication (quorum quenching, QQ). A systematic study was performed with α-, β-cyclodextrin, and their random methylated, quaternary amino and polymer derivatives to evaluate and compare their effects on the biofilm formation of *Pseudomonas aeruginosa*. To examine the concentration-, temperature- and time-dependency of the QQ effect, the CDs were applied at a 0.1–12.5 mM concentration range, and biofilm formation was studied after 6, 24, 48 and 72 h at 22 and 30 °C. According to the results, the QS mechanism was significantly inhibited; the size of the cavity, the structure of the substituents, as well as the monomeric or polymeric character together with the concentration of the CDs have been identified as key influencing factors of biofilm formation. Statistically determined effective concentration values demonstrated outstanding efficiency (higher than 80% inhibition) of α-CD and its random methylated and polymer derivatives both on the short and long term. In summary, the potential value of CDs as inhibitors of QS should be considered since the inhibition of biofilm formation could significantly impact human health and the environment.

## 1. Introduction

Amongst several infectious mechanisms of bacteria, biofilm formation is thought to determine 65–80% of all microbial infections [1]. *Pseudomonas aeruginosa* is an opportunistic pathogen with the ability to form biofilm, causing acute and chronic infections [2]. One of the most relevant regulation systems of *P. aeruginosa* associated with biofilm formation is the quorum-sensing mechanism [3], which can be controlled by inhibiting agents, such as the water-soluble cyclic oligosaccharides, the cyclodextrins [4]. Biofilms consist of microbial cells attached to various surfaces, including living tissues, piping of industrial or potable water systems, indwelling medical devices, and natural aquatic systems. Cells in the biofilm are surrounded by a self-produced extracellular polymeric substance (EPS) matrix, which constitutes their immediate environment [5].

The EPS has several functions, including offering a stable environment for microbial cells, supporting their adaptation to stressors and coordination of activities in response to changes triggered by the environment [6].

Biofilms in natural or engineered environments may constitute both a problem and a solution in engineering practice [6,7,8,9]. The adverse effects of biofilm formation (biofouling, biocorrosion) on the surface of various solid materials may cause serious problems in wastewater treatment, metalworks, and food processing [10]. In addition, biofilm formation is an important mechanism of developing bacterial resistance, leading to difficulties in controlling bacterial infections in humans and animals, since biofilms are highly resistant to antibiotics and the host immune system [7,11]. It has been recognized that biofilm formation determines 65–80% of all microbial infections amongst several bacterial infectious mechanisms [1,12].

Bacteria with the ability to form biofilms are under the control of the quorum sensing (QS) system [13,14]. Quorum sensing, originally described in the early 1970s for the Gram-negative marine bioluminescent bacterium, *Aliivibrio fischeri* [15], constitutes a cell-to-cell communication process among bacteria through the production and release of extracellular signals termed autoinducers. Both Gram-positive and Gram-negative bacteria use this bacterial communication network to influence the expression of virulence factors and biofilm formation, but also bioluminescence, toxin production, motility, exopolysaccharide production, etc. [15,16,17]. According to the QS mechanism, bacteria communicate, cooperate, and perceive the cell population density and respond to the information by controlling gene expression [16]. The QS system has the following components: the QS signal synthase, the signal receptor, and the signal molecule. The signal molecules function as local sensors to communicate population densities in bacteria [18]. As the bacterial population grows, the number of signal-receptor complexes reaches a threshold concentration, activating the expression of target genes [18] that encode the special phenotypical properties mentioned above [19]. Gram-negative bacteria produce various N-acyl-L-homoserine lactones (AHLs) as signal compounds, while the Gram-positive bacteria produce signal peptides called autoinducing peptides (AIPs) [20,21,22,23,24].

*P. aeruginosa*, a Gram-negative, opportunistic, pathogenic bacterium commonly occurring in soil and water, is capable of forming biofilm. This bacterial species expresses various cell-associated or extracellular quorum-sensing-controlled virulence phenotypes [2]. It is known to infect a broad range of hosts, including humans, plants, and animals, and therefore is responsible for both acute and chronic infections, particularly in individuals with airways diseases as well as immunocompromised, burned, or wounded patients [25,26]. The AHL-mediated QS network in *P. aeruginosa* is made up of four QS systems, namely the LasI/LasR and the RhlI/RhlR systems [27], the PqsABCDE/PqsR system [28], and the AmbBCDE/IqsR system [29]. Each system has its own signal molecule: 3-oxododecanoyl-L-homoserine lactone (3-oxo-C12-HSL), N-butanoyl homoserine lactone (C4-HSL), 2-heptyl-3-hydroxy-4-quinolone (Pseudomonas quinolone signal—PQS) and 2-(2-hydroxyphenyl)-thiazole-4-carbaldehyde (integrated quorum-sensing signal—IQS), respectively [3]. These QS systems are hierarchically related and interact with each other [30]. Meanwhile, these systems co-regulate the expression of various genes related with motility, biofilm formation, immune evasion, iron scavenging, and antibiotic resistance [3,31,32].

The inhibition of the processes involved in the control of QS is called quorum quenching (QQ) [33]. Biofilm-related pollution causes serious problems in many fields, such as health [34,35,36], environmental science [37], wastewater treatment, and engineering [38,39]. For this reason, the engineered control of QS to inhibit undesired microbial activities is a promising preventive strategy for QS-mediated cell functions, including biofilm formation and virulence factor production in infectious diseases, etc. [14]. The QS-inhibiting agents may include both natural bioactive molecules produced by prokaryotes and eukaryotes, as well as synthetic molecules or compounds, as analogues of signal molecules [40]. QS inhibiting agents control biofilm formation by targeting the QS signal molecules or their receptors, or downstream regulatory factors [14,38], and thus, they interfere with the expression of virulence factors and suppress biofilm formation.

The QS-inhibiting agents targeting the QS signal molecule may be involved in the inactivation of the signal molecule synthases, neutralization of AIPs with antibodies, modification, or degradation of the signal molecules, etc. [14,34]. By targeting the receptors of the QS signal molecules, the QS-inhibiting agents may inactivate the receptor or compete for the receptor to prevent virulence factors expression and biofilm formation [41,42]. One of the QQ mechanisms targeting the QS signal molecule is sequestration of the AHLs by host materials to disrupt signaling [43,44]. To control virulence factors, as well as biofilm formation in *P. aeruginosa* model system, several natural and artificial QS inhibitors (parthenolide, flavonoids, 3-phenyllactic acid, sitagliptin, naringenin, furanones, ureidothiophene-2-carboxylic acids, etc.) were studied and evaluated [36,40,41,42].

Control of QS by cyclodextrins (CDs) is an innovative approach; the available information about their effects on bacterial communication regulated processes is limited. The comparative effect of various cyclodextrin derivatives on biofilm formation in the *P. aeruginosa* model system, function of concentration and time, had not been assessed so far in a systematic study.

Cyclodextrins (CDs), the water-soluble cyclic oligosaccharides, which possess a hydrophilic external surface and a hydrophobic internal cavity [45,46,47], proved to be suitable host materials for inclusion of the AHL signals with different lengths of the acyl chain by hydrophobic interaction in aqueous media [44]. CDs have the capability to encapsulate hydrophobic guests, forming inclusion complexes with a range of organic compounds [46]. Due to the complex-forming capability of CDs, which could increase the solubility, bioavailability, and stability of the included compounds, they have been widely used in drug formulation [47,48], cosmetics, and the food industry [49], as well as in environmental science [50]. In addition, it was demonstrated that CDs interacted with N-hexanoyl homoserine lactones, the quorum-sensing signals produced in Gram-negative bacteria [4,43,44,51]. The widely applied molecules differing in cavity size are the native α-, β- and γ-cyclodextrins (ACD, BCD and GCD), and their highly water-soluble derivatives [52]. Some of these derivatives are industrially produced for various applications, while others have been regularly synthesized in our laboratory for own studies or to be sold as fine chemicals. The large palette of these derivatives made it possible to select the most promising ones to be studied in detail.

Some studies focused on the effect of cyclodextrins on AI-mediated QS parameters in *P. aeruginosa*, or the investigation of the QS pathways and QQ possibilities in other bacterial model systems such as *Serratia marcescens*, *Chromobacterium violaceum*, or *Aliivibrio fischeri* [51,53,54,55]. Ikeda et al. [4] presented the first approach to control quorum sensing in Gram-negative bacteria by adding host compounds of signal molecules. First, 10 mM cyclodextrin or glucose was added to the *P. aeruginosa* bacterial culture medium and β-galactosidase activity was determined. Molnar et al. [51] demonstrated that the autoinducer-dependent quorum sensing mechanism in the *A. fischeri* model system, focusing on bioluminescence, was markedly inhibited through the QQ effect of twelve cyclodextrins. Morohoshi et al. [55], designed and synthesized a series of novel CD derivatives to improve the QS inhibitory activity over that of native CDs. The QQ efficiency of the native cyclodextrins and their various derivatives was examined, focusing on the elastase production of *P. aeruginosa* PAO1, the prodigiosin production of *S. marcescens*, and violacein production of *C. violaceum*.

Although numerous potential QS inhibitors were studied to influence the QS-mediated virulence factors in *P. aeruginosa* [56,57,58,59,60], there is only scarce information on the effect of various CDs on quorum sensing and even less on biofilm formation. The available papers on *P. aeruginosa* QS model system reported valuable information on the effect of some native CDs, derivatives, or immobilized engineered systems on QQ [4,53,54,55,61,62], but extensive, comparative studies on a wide range of CD derivatives in a time- and concentration-dependent manner, focused specifically on biofilm formation, were not published.

The main aim of this study was to test and evaluate the potential concentration- and time-dependent quorum-quenching (QQ) ability of selected cyclodextrin molecules, which might interfere with the control mechanisms of biofilm formation by *P. aeruginosa* without directly affecting bacterial viability. In support of this concept, the cytotoxic effect was also monitored further to the effect of the studied CDs on the QS system. In addition, we planned to develop and apply a simple and high-throughput method for quantitative characterization of QS-mediated biofilm formation in *P. aeruginosa*, which allowed sensitive and reliable detection of the effect of cyclodextrins.

Modulation of the QS control mechanisms of biofilm formation of *P. aeruginosa* with such a wide range of CDs and the systematic approach is unique in the scientific literature.

## 2. Results

### 2.1. Effect of Cyclodextrins on Biofilm Formation of Pseudomonas aeruginosa

The potential quorum quenching effect of α- and β-CD (ACD, BCD), randomly methylated α- and β-CD (RAMEA, RAMEB), quaternary ammonium α-CD and β-CD (QAACD, QABCD), and α- and β-CD polymers (ACDPS, BCDPS) on the biofilm formation capacity of *P. aeruginosa* was tested within the 0.01–12.5 mM concentration range in a small volume (200 µL) model system applying 96-well microtiter plates.

**Figure 1:**
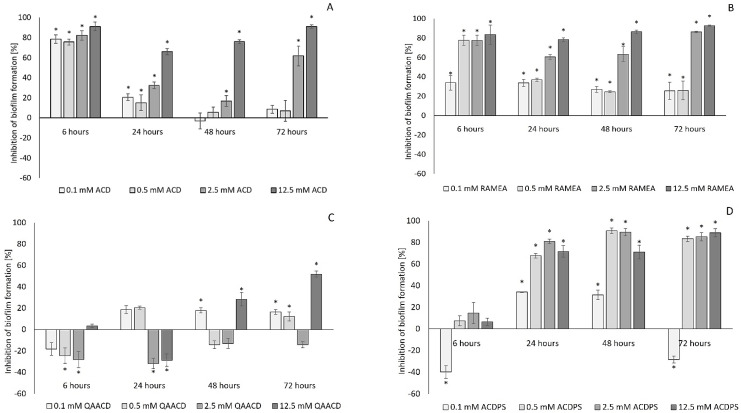
Effect of increasing concentrations of ACD (**A**); RAMEA (**B**); QAACD (**C**); and ACDPS (**D**) on biofilm formation at 22 °C. Significant inhibition compared to control is marked by asterisk (*) (*p* < 0.05). Data represent averages of five replicates.

**Figure 2:**
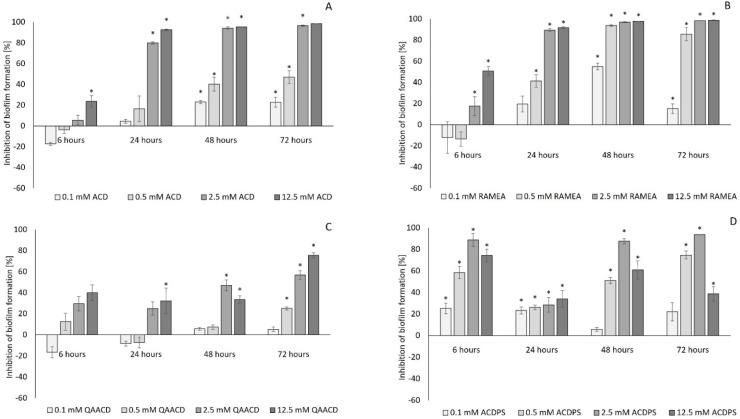
Effect of increasing concentrations of ACD (**A**); RAMEA (**B**); QAACD (**C**); and ACDPS (**D**) on biofilm formation at 30 °C. Significant inhibition compared to control is marked by asterisk (*) (*p* < 0.05). Data represent averages of five replicates.

As expected, the native ACD and its tested derivatives significantly affected the biofilm formation both at 22 °C (Figure 1) and 30 °C (Figure 2). The degree of inhibition compared to the control ranged from 0 to 99% depending on the type, concentration, contact time of the cyclodextrin, and the incubation temperature.

As illustrated by the figures (Figure 1 and Figure 2), the extent of inhibition was significantly influenced by the cyclodextrin concentration. Generally, the inhibition was greater at the higher incubation temperature (30 °C). The increase in the CD-concentration resulted, generally, in higher inhibition values, except for QAACD, where there was no clear relationship between the tested cyclodextrin concentration and the degree of inhibition.

**Figure 3:**
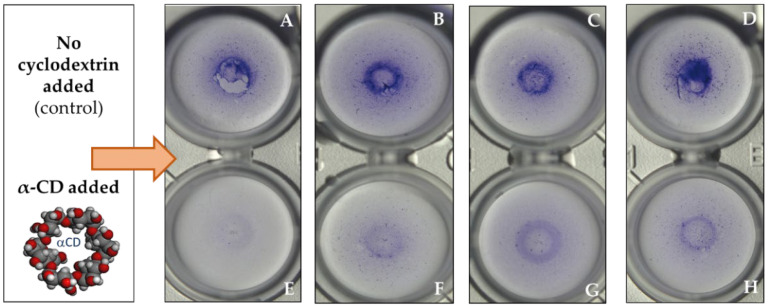
Light microscopy images of the *P. aeruginosa* biofilm at 48 h-incubation dyed with 0.1% crystal violet, in presence of distilled water (**A**–**D**); or 12.5 mM (**E**); 2.5 mM (**F**); 0.5 mM (**G**); 0.1 mM (**H**) α-CD.

The native α-CD, its randomly methylated derivative, and the α-CD polymers exhibited concentration-dependent inhibition; the inhibitory effect was already detectable after 6 h, and it kept on growing significantly for 72 h. Especially high and significant inhibitory effect (90–98%) was found for ACD and RAMEA at 12.5 mM concentration. It is noteworthy that ACD resulted in outstanding inhibition of biofilm formation (~78%) at as low as 0.1 mM concentration after 6 h, which decreased at the 22 °C series in the later sampling points. Light microscopy images (Figure 3) illustrate the crystal violet stained biofilm formed in the wells of the microplates. There is a clear decrease in biofilm formation due to treatment with ACD at different concentrations.

The results of repeated measures variance analyses (RMANOVA) of ACD and ACD derivatives (Table 1 and Table 2) demonstrated the significant efficiency of ACDs; both the contact time and the cyclodextrin treatments influenced the biofilm formation capacity of the bacteria. The effect of CD treatments was also significantly different in time, with the exception of QAACD at 30 °C.

In respect of the optical density values (Supplementary Materials S1 Tables S1–S8), generally there was no significant decrease triggered by cyclodextrins compared to the control. Only 12.5 mM ACD caused more than 20% inhibition (~33%) of the optical density of the whole cell suspension (planktonic phase and biofilm) after 6 h at 22 °C. Otherwise, the CDs had stimulating effects in most cases.

According to the results of the OD measurement of the planktonic supernatant, inhibition was observed in several cases. Mainly for ACD and RAMEA at 6 h contact time, the inhibition was 44–79% and 10–35%, respectively. Moreover, ACDPS-treatment resulted in significant decrease in OD after 24 h (20–28%, at 22 °C, and 16–49%, at 30 °C). However, the OD of the planktonic supernatant was primarily stimulated by CD, with the exception of ACD and RAMEB. Thus, this might indicate that none of the tested CD derivatives had a high detrimental effect on the cells. However, it is important to note that the OD is proportional to the total number of cells in which both living and dead cells are considered; therefore, correct conclusions on cytotoxicity can be drawn from these data only with great caution or not at all.

For this reason, the possible cytotoxic effect of cyclodextrins was also determined using the resazurin reduction assay in order to assess the potential detrimental effect of CDs on the viability of the cells, which might influence the QS process as well. The resazurin reduction assay characterizing enzyme activities (e.g., reductases, dehydrogenases) is widely used in viability tests. The formation of a water-soluble, fluorescent product by metabolically active cells during the assay is the primary advantage of this test compared to the tetrazolium reduction methods. Despite their sensitivity, they also have drawbacks, as several factors (e.g., temperature, pH, and resazurin concentration) may affect the result [63]. Thus, considering all these, we also found that in most cases, only the results measured at 22 °C incubation temperature could be used reliably to characterize viability in the tested period of time (72 h).

According to the results, generally, the ACD and its derivatives did not show inhibition. Only the highest tested concentration (12.5 mM) of ACD and RAMEA at 72 h contact time resulted in 40 and 55% inhibition in the viability of *P. aeruginosa*, as demonstrated by the resazurin reduction assay at 22 °C. However, we found that the measurements at 30 °C incubation using the resazurin reduction method did not give reliable results, and sensitivity was inadequate.

**Figure 4:**
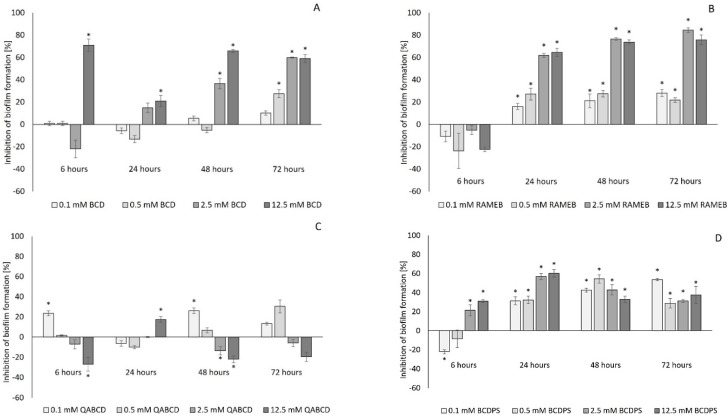
Effect of increasing concentrations of BCD (**A**); RAMEB (**B**); QABCD (**C**); and BCDPS (**D**) on biofilm formation at 22 °C. Significant inhibition compared to control is marked by asterisk (*) (*p* < 0.05). Data represent averages of five replicates.

**Figure 5:**
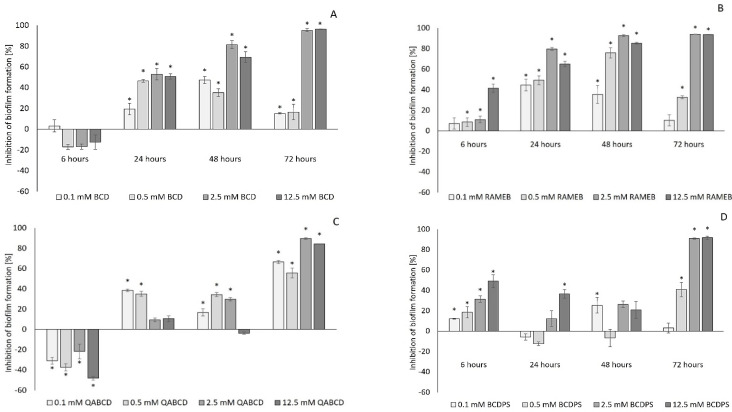
Effect of increasing concentrations of BCD (**A**); RAMEB (**B**); QABCD (**C**); and BCDPS (**D**) on biofilm formation at 30 °C. Significant inhibition compared to control is marked by asterisk (*) (*p* < 0.05). Data represent averages of five replicates.

Figure 4 and Figure 5 show the effect of BCD and BCD derivatives on the biofilm formation of *P. aeruginosa*. The QQ effect was significantly affected by the cyclodextrin concentration, similarly to ACD and its derivatives; however, the degree of inhibition was generally lower than that of the corresponding ACD, RAMEA, QAACD, and ACDPS. The highest inhibition (~96%) was achieved by 12.5 mM BCD after 72 h. Among the studied BCDs, QABCD exhibited the lowest quorum quenching effect at 22 °C of incubation, similarly to ACDs. As illustrated, the highest inhibition caused by QABCD was lower than 30%. The results clearly demonstrated that the RAMEB derivative generally mediated significantly higher inhibition compared to the native BCD.

Regarding the influence of the incubation temperature, generally, the inhibition rate of BCDs was greater at the higher incubation temperature (30 °C), similarly to the ACD and its derivatives.

For the optical density values of BCDs (Supplementary Materials S1 Tables S9–S16), generally there was no significant decrease caused by cyclodextrins compared to the control, except for one case. QABCD caused 22–44% inhibition in the optical density of the population after 6 h, depending on the concentration. Except for this, based on OD measurement, only stimulatory effects were observed with beta-cyclodextrins in the planktonic phase too. According to these results, the quorum quenching effect of BCDs was mostly obvious. Meanwhile, in some instances based on the resazurin assay, mainly at longer contact times (48 h or 72 h) and at the highest tested concentrations, the BCDs had an inhibitive effect on viability (Table 3).

The highest inhibition (~96%) was achieved by 12.5 mM BCDPS after 72 h; however, significant cytotoxic effect (38%) was observed after 48 h as well. These results clearly indicate the cytotoxic effect, which may interfere with the QQ effect of these CDs.

The RMANOVA analysis of BCD and its derivatives (Table 4 and Table 5) supported our observations regarding the effect of cyclodextrin: the treatments significantly influenced the formation of bacterial biofilms. At the same time, the effect of treatments differed significantly in time.

### 2.2. Efficiency of the Cyclodextrin-Mediated Effects on Biofilm Formation Based on Their Effective Concentration Values

For comparative evaluation of the effect of various cyclodextrins, effective concentrations resulting in 10, 50, and 90% inhibition were determined by statistical analysis based on their concentration-response analysis. EC_10_, EC_50_, and EC_90_ values are summarized in the following figures (Table 6, Table 7 and Table 8).

According to the EC_10_, EC_50_, and EC_90_ values in Table 6, Table 7 and Table 8, the more effective treatments are indicated in red (and its shades), while green and its shades show less effective cyclodextrin applications. The lower this value, the more effective the cyclodextrin in reducing biofilm formation. In some cases, when the concentration–response relationship could not be studied by the software, it was not possible to determine the EC values (indication: not determined—n.d.).

A concentration that achieves 10% effect on the measured endpoint is a good indicator for the lowest effective concentration of cyclodextrins providing significant inhibition. Based on the lowest EC_10_ values, the most effective cyclodextrins are ACDPS, RAMEA, and RAMEB. ACDPS effectively inhibited biofilm formation even at very low (0.01 mM, 24 h, 30 °C) concentrations. The effectiveness of a drug or any chemical in the biological/physiological processes of the organisms is commonly quantified as EC_50_, the concentration that leads to 50% maximal response. Effective concentrations causing 50% inhibition illustrated high efficiency of RAMEA, too.

Comparing the EC_90_ values (Table 8), the different degrees of cyclodextrin-mediated efficiencies on biofilm formation could be also characterized. The 90% effective concentration (EC_90_) provides a more sensitive measure and a practically usable endpoint of the efficiency of cyclodextrins than the EC_50_ value. Ninety percent inhibition was achieved only in a few cases, the highest at 30 °C for ACD and RAMEA, which was a good indication of the outstanding efficacy of these two cyclodextrins.

The data in the three tables clearly illustrate the greater efficacy of ACD and its derivatives versus the effect of BCD and its derivatives. Additionally, the results reflect the higher inhibitions obtained at higher incubation temperatures (30 °C).

## 3. Discussion

Since biofilm formation is one of the most important virulence factors of the opportunistic pathogen species, *P. aeruginosa*, biofilm production and its influencing parameters have been thoroughly investigated using different *P. aeruginosa* strains [2,3,30]. As a quorum-sensing (QS)-regulated process, microbial biofilm formation is one of the major causes of bacterial infections, triggering problems not only in two key areas such as biotechnologies and human health. Different innovative approaches have been used to destroy and eliminate the threat of biofilms, one of which is the use of cyclodextrins to influence this QS-driven process.

In this study, an extensive series of experiments were performed to examine the effect of various cyclodextrins—including native molecules, their randomly methylated-, trimethyl-aminopropyl-derivatives and epichlorohydrin-crosslinked polymers—on the QS system of *P. aeruginosa* PAO1. These derivatives were selected based on our previous experience with the bioluminescence studies of *Aliivibrio fischeri* [51]. Introducing hydrophobic methyl groups to the randomly methylated derivatives resulted in an extended cavity size which proved to be usually advantageous for complex formation with the hydrophobic guest molecules. Introduction of a positive charge was expected to have antibacterial effect due to the interaction with the negative surface of bacteria, improving the QQ effect of the cavity. In case of polymer derivatives, the cooperative action of the neighboring cavities was expected to result in enhanced inhibition of biofilm formation.

The main objective of our study was the time- and concentration-dependent effect assessment of cyclodextrins on the biofilm formation capacity of *P. aeruginosa*. We also aimed to test the applicability of a previously described assay (O’Toole’s methodology) and its possible development for the sensitive, reliable, and routine monitoring of biofilm production. Testing the applicability of a new QS inhibitor requires extensive research and a systematic approach. Therefore, high-throughput procedures are necessary to establish such a unique methodology.

### 3.1. Evaluation of the High-Throughput Microtiter Plate Assay Applied for Quantification of Biofilm Formation

This research also demonstrated the applicability of the microtiter plate assay as a high-throughput screening tool for characterizing the influence of cyclodextrins on the biofilm formation capacity of *P. aeruginosa*. The method applied in this study was a microtiter plate assay based on O’Toole’s methodology [64]. The original methodology aims to quantify biofilm by staining the biofilm with crystal violet, then solubilizing the dye bound by the biofilm, and then measuring the absorbance spectrophotometrically. This method with adequate sensitivity is suitable for the simultaneous examination of numerous samples in several replicates; however, the original approach has a few drawbacks. To improve the applicability of the method, several parameters were varied to find the best adjustment for measuring population growth, intensive biofilm formation, and the most effective routine examination of the effect of various cyclodextrins. The M63 growth medium suggested by O’Toole [64] and the Luria–Bertani (LB) medium were both tested in the optimization process in the preliminary experiments (data not shown). The LB medium proved to be more suitable for testing the effect of cyclodextrins on the QS system; the growth of bacteria was more intensive, and the matrix was thicker and more structured.

Furthermore, we extended the contact time of the O’Toole’s microtiter biofilm assay.

The 4–24 h (suggested in the original methodology) or the 24–48 h exposure time (tested in the preliminary experiments) do not seem long enough to examine the construction and degradation phases of the biofilm and to follow both the short-term and the long-term effect of cyclodextrins in the test system. For this reason, the exposure time was extended to 72 h and the quantification of the biofilm was performed after 6, 24, 48, and 72 h of incubation. The extended monitoring period gave outstandingly good and exciting results. According to our observations, some cyclodextrins (ACD, RAMEA) resulted in extremely high inhibition even after 6 h. In comparison, some other derivatives had a significant effect on biofilm formation only after 72 h, which justifies monitoring the biofilm at different time intervals, mainly when testing the impact of a new QSI.

One of the most critical factors in the biofilm formation experiments was the incubation temperature; the original methodology suggests 37 °C. Still, we experienced that at this temperature, the biofilm formation was very intensive, and the growth of the population reached the death phase quickly. However, considering the practical realization of biofilm formation, 22 and 30 °C were relevant, so we performed our experiments at these two temperatures. According to the results, both incubation temperatures were appropriate for biofilm formation. Although at 22 °C, the matrix was thinner, and the biofilm formation and the growth of the bacterial population were slower, we found that certain stages of the process could be observed better at lower temperature. As a result, both 22 and 30 °C were applied as incubation temperatures.

Additionally, the separation of the planktonic phase and the analysis of its viability were introduced. The critical step of the original methodology was the removal of the remaining test medium and of the planktonic cells (after incubation). We found that even the thickest, most stable biofilm was severely damaged during these cleaning steps; therefore, we transferred the supernatant to the same wells of a new 96-well microtiter plate, taking care of the integrity of the biofilm formed on the cell wall and on the surface of the liquid. The remaining test medium was removed from the cells as described by O’Toole [64]. We found that the introduction of this step decreased the damage to the biofilm, increased the sensitivity of the method, and in addition, cell vitality could be tested in the supernatant based on the resazurin reduction method described by Palomino et al. [65].

### 3.2. Quorum Quenching Effect of Cyclodextrins

This in vitro study demonstrated significant inhibition of *P. aeruginosa* biofilm formation with cyclodextrins. The type and concentration of cyclodextrins influenced the degree of inhibition of biofilm formation. Inhibition might be due to several mechanisms, such as the quorum quenching and the potential cytotoxic effect of cyclodextrins, which was studied by two methods (see Section 3.3), and it could be the outcome of the QSI-mediated attenuation of biofilm formation. The interference into the QS pathway to silence bacterial communication could be achieved with QS-inhibiting agents, including mainly QS inhibitors (QSIs) and quorum-quenching (QQ) enzymes [14]. Some investigations have demonstrated that the acyl chains of the AHLs can be included into the hydrophobic cavity of CDs in an aqueous solution, thus controlling QS [43,44,53,54,66]. However, the native CDs proved to have lower QS inhibitory effects than other natural and artificial inhibitors [53,54,55].

Our research clearly revealed that both the native ACD and its random methylated and polymer derivative were highly efficient, exhibiting more than 90% inhibition of biofilm formation at a 12.5 mM concentration level.

Pearson product moment correlation analysis demonstrated that the added ACD, RAMEA, and ACDPS considerably (proportionally with the concentration) reduced the cell-to-cell communication (Supplementary Materials S2). Very strong negative correlation (−0.98, at 48 h) was found between ACD concentration and biofilm formation (at 22 °C) and RAMEA at 30 °C (−0.88 at 6 h), at both the shorter and longer contact time, indicating the outstanding quorum-quenching capability of ACD and RAMEA (Supplementary Materials S2 Tables S1 and S2).

Although RAMEB was also found to be more effective than native BCD, the effect of these cyclodextrins was much smaller than that of ACD and its derivatives. This may be related to the cavity size [67]; presumably, the ACD with its smaller cavity size is able to form more stable complex with the acyl chain of the signals.

The highest efficacy was achieved with RAMEB, which could be associated with the fact that the randomly methylated derivatives had better water solubility [67]. The biological activity of cyclodextrins may also be affected by their aggregation behavior [68]. The physical state of dissolved cyclodextrins is of paramount importance, especially when studying their properties in biological systems. The aggregation performance may influence the efficiency of CDs in biological systems [68]. The lower efficacy of BCDs in our study may be due to the formation of aggregates. Both Coleman et al. [69] and Loftsson et al. [70] observed that β-CD might form aggregates, which is a concentration-dependent process. The aggregate formation effect and the partial substitution is less pronounced in case of ACD [71].

According to Ikeda et al. [4], an inhibitory effect was observed on the autoinducer activities of the quorum sensing in *P. aeruginosa* by the addition of α-CD, β-CD, dimethyl-β-CD, and trimethyl-β-CD, because they could form a complex with signal molecules, while γ-CD showed no effect on AI activity. None of the additives showed an effect on the growth of bacteria, contrary to our results with ACD and RAMEA, showing a slight cytotoxic effect. However, methods for determination of bacterial growth were not presented in Ikeda et al.’s study [4].

According to Kato et al. [53], CD-immobilized polymer gel sheets could regulate QS by effectively controlling the prodigiosin production in *S. marcescens*. The relative prodigiosin production of *S. marcescens* in the presence of 10 mM 2-hydroxypropyl-β-CD (HPBCD) could be reduced to approximately 86% compared to the control. The addition of both anionic carboxymethyl cellulose gel sheets and 10 mM HPBCD effectively controlled the relative prodigiosin production to approximately 0.56.

Okano et al. [43], investigated the effect of HPBCD on the prodigiosin production of *S. marcescens*. The relative prodigiosin production decreased with the increase in cyclodextrin addition so that it was almost blocked at 12% of HPBCD, showing that the β-CD cavities could capture the acyl chain of the AHLs and could interact for QS inhibition. They also examined the QS inactivation by a bioassay of an AHL-synthase defective mutant strain of *C. violaceum* (CV026) and found that 20% HPBCD induced a meaningful reduction of violacein production after 10 h of exposure.

Morohoshi et al. [55] investigated the influence of alkylamine-modified cyclodextrins on QS-mediated processes in different bacterial model systems (*C. violaceum*, *S. marcescens* and *P. aeruginosa*). Although native β-CD did not show any inhibitory activity on elastase production of the *P. aeruginosa* PAO1 strain, the 2-alkylamino-CDs and 6-alkylamino-β-CD derivatives reduced the QS-mediated elastase production (of *P. aeruginosa* by approximately 20%, [59]). The synthesized CD derivatives also had strong inhibitory effects on the QS of *C. violaceum* and *S. marcescens*.

During the last years, novel types of engineered QS inhibitory materials were developed involving various CDs for preventing QS in various bacterial model systems. Miller et al. [72] used silicon dioxide nanoparticles (Si-NPs) surface-functionalized with BCDs to reduce cell-to-cell communication in an *A. fischeri* model system through binding of AHLs to the nanoparticles, thus removing them from the immediate bacterial environment.

Okano et al. [44] demonstrated QS inhibition by applying BCD-modified microspheres, monitoring the prodigiosin production in *S. marcescens* AS-1 and pyocyanin production in *P. aeruginosa* AS-3. Production of prodigiosin was reduced to approximately 72% in the presence of 5mM immobilized BCD, while pyocyanin production was reduced to 40%.

Takayama and Kato [73] have shown that the immobilized ACD and HPBCD on HPC/alginate gel fibers could inhibit the C6-HSL-mediated prodigiosin production to 10% in the opportunistic human pathogen *S. marcescens* AS-1.

Molnar et al. [51] monitored the concentration- and time-dependent bioluminescence inhibitory effect of twelve CDs in the *A. fischeri* model system. The efficiency was proved to be influenced by the size of the interior cavity, the structure, and the concentration of the cyclodextrins, as well as the contact time with the cells.

Although the above studies illustrated the potential efficacy of cyclodextrins in inhibiting the QS processes, they generally tested the effect using only one concentration at a time and generally achieved less efficacy than the current study applying ACD, RAMEA, and ACDPS. This research constitutes a novelty in terms of characterizing the time-, temperature-, and concentration-dependent QQ effects and efficiency of various cyclodextrins and cyclodextrin derivatives on the biofilm formation of *P. aeruginosa*.

There may be a number of mechanisms behind time-dependent effects. The exact mechanism and background of the QS-stimulating effect is not known yet, especially in the presence of cyclodextrins. With time, the inhibitory effects of CDs may change through variation of the QS regulation processes at different growth phase of biofilm formation, resulting in the time-dependent phenomenon.

*Pseudomonas aeruginosa* uses hierarchical complex quorum-sensing systems for the regulation of biofilm formation [30]. The two well-studied QS systems, the LasI/LasR and the RhlI/RhlR systems, co-regulate the expression of various genes related to biofilm formation. Each system has its own signal molecule: 3-oxododecanoyl-L-homoserine lactone (3-oxo-C12-HSL) and N-butanoyl homoserine lactone (C4-HSL), respectively. In addition, the RhlI/RhlR QS system is under the regulatory control of the LasI/LasR system. The RhlI/RhlR system regulates the expression of rhamnolipids, which may have a key role in late-stage biofilm formation.

Since cyclodextrin is thought to complex the acyl chain of the signaling molecules [43,44,51], neither the LasI/LasR system nor the RhlI/RhlR QS system will be activated in the presence of CD. Due to complexation by CD, the available concentration of the signal molecules will be much lower than that of the control. Thus, in the presence of an appropriate cyclodextrin concentration, biofilm formation may be inhibited for a longer period of time, whereas biofilm formation will be increased in the untreated control. As a result, the inhibition level will grow in the system over time. This argument was also supported by the fact that the time-dependent inhibitory effect of cyclodextrins was less common at low concentrations. The possible cytotoxic effects of cyclodextrins may increase this inhibitory effect as well.

Upon binding the signal molecules, cyclodextrins may change the transition efficiency from the inactive to the active form. Since CDs may affect the processes regulated by both signal molecules (3-oxo-C12-HSL and C4-HSL), this can result in different effects and different degrees of inhibition over time. Thus, with time, the effects of CDs may change according to the variation in the QS regulation processes at different growth phases, resulting in the time-dependent phenomenon.

Despite the complex QS-systems of *P. aeruginosa* in terms of activation, regulation, and interaction of biofilm-encoded genes, the findings of this study strongly suggest that cyclodextrins could be a potential candidate for QS inhibition as an antivirulence compound.

### 3.3. Cytotoxicity of Cyclodextrins

Our previous research has already demonstrated the slight cytotoxic effect of ACD [51]. Although cytotoxicity may decrease the biofilm formation capacity, only a few researchers examined viability as a complementary method for monitoring QS-driven processes. Even though the inhibition of viability is generally described based on optical density (OD) measurements [55], OD is not an appropriate indicator, as the OD value is proportional with the total cell number. The viability of the cells in this study was tested by measuring the optical density of both the whole bacterial test medium and the planktonic supernatant.

Although the different redox methods may have several disadvantages, they are more closely related to the number of living cells. In the preliminary experiments of our research (data not shown), we studied the applicability both of the MTT test based on tetrazolium reduction and of the resazurin assay based on resazurin reduction, but regarding the flexibility and sensitivity, the resazurin-based assay proved to be better at room temperature. Thus, to test viability in this study, an optimized resazurin reduction method was applied, which, according to the literature, has not been used so far in biofilm formation experiments. In spite of this, based on the results, this method did not show relevant results at 30 °C incubation for more than 48 h, presumably due to the pH change [74]. Furthermore, the results of the resazurin test might be influenced by the presence of CDs, as shown by Csepregi et al. [75] via (1) inhibition of the cellular uptake of resazurin and (2) enhancement of the fluorescence signal of the formed resorufin.

According to the results of the cytotoxicity tests, in most cases, the ACDs did not show high inhibitory effect, so the effect of cyclodextrins could be considered the primary effect mechanism of the QS process. However, ACDPS and RAMEA in some cases had adverse effects on viability, mainly at longer contact times (72 h) and at the highest concentrations (12.5 mM) tested. In spite of this, with one exception (12.5 mM RAMEA, 72 h, 55%), these inhibitory effects did not exceed 40% compared to controls. At the same time, it is noteworthy, that these two cyclodextrins had no cytotoxic effect at lower tested concentrations (<12.5 mM) or other contact times.

Regarding the effects of BCDs on viability, slight inhibition was observed in some cases, mainly at 12.5 mM concentration of these CDs, and at longer contact time (48 and 72 h). Previous results discussing the effect of cyclodextrins on human health were summarized by other authors [76,77]. They found that BCD and methyl-BCD induced apoptotic cell death in human keratinocytes because of their affinity for membrane cholesterol and its esters. According to Stella and He and Kiss et al. [76,77], there was a positive correlation between the hemolytic activity of several CDs and their capacity to solubilize cholesterol. The authors concluded that although native ACD and BCD and their alkylated derivatives were disruptive of biological membranes, GCD, HPBCD, and SBEBCD as well as other non-cholesterol-solubilizing CDs appeared safer for human use.

Currently, the available knowledge about the potential cytotoxic effect of cyclodextrins on prokaryotic cells is limited. The previous experiments mainly focused on human cells and the high affinity of cyclodextrins for membrane-forming cholesterol, but their results are less valid for bacteria due to the differences in cell structures and cell membranes. However, Aachmann and Aune [78] demonstrated that CDs may affect the bacterial uptake of DNA by interacting directly with the cell wall.

It is likely that CDs can extract membrane compounds, thus making it more permeable for DNA. It was hypothesized that the CDs extract and make inclusion complexes with lipids and other hydrophobic moieties from the cell membrane, which become more permeable, thus facilitating the uptake of DNA into the bacterial cell. The mild and medium-level cytotoxic effects observed in our research may also be due to this mechanism, but this needs to be tested in further membrane permeability studies.

### 3.4. Future Research Directions

Although it has been clearly demonstrated that cyclodextrins can influence QS-driven processes, there are still many unanswered questions.

In the future, it is recommended to test the effect of CDs on biofilm formation by adding signaling molecules in different combinations with cyclodextrins, an approach which proved to be effective in our previous studies [51]. Furthermore, we plan to determine the complex association constants of signaling molecules with the selected cyclodextrins. One of our long-term goals is to explore and efficiently influence the QS-controlled processes in bacterial systems working with more than one signal, applying a combination of cyclodextrins.

There is a need for further experiments to assess the mechanism of ACD-, RAMEA-, and ACDPS-mediated high efficiency in QS. The emphasis should be on exploring the background mechanisms of the cytotoxic effects, on increasing the efficiency of CD-induced quorum quenching, as well as on assessing the CD structure-dependent effects.

Ultrastructural characterization of the biofilm matrix and its embedded bacterial cells is also planned, and the study of the effect of CD-treatments on biofilm formation by scanning electron microscopy with customized protocols.

## 4. Materials and Methods

The time- and concentration-dependent effect of native α- and β-cyclodextrins (ACD and BCD), their monomer derivatives, as well as the epichlorohydrin-crosslinked polymers on the biofilm formation and viability of *P. aeruginosa* PAO1 was tested in a series of experiments at 0.1–12.5 mM concentration range. The systematically examined cyclodextrins of various structures were all fine chemicals of CycloLab Cyclodextrin R & D Laboratory Ltd.

To differentiate between the quorum-quenching effect and the cytotoxic effect of the cyclodextrins, the optical density (OD) and the resazurin reduction activity (RRA) were also determined. The effect of temperature on biofilm formation, as well as the efficiency of cyclodextrins, was studied at two different temperatures (22 and 30 °C).

### 4.1. Bacterial Strain and Culture Conditions

The bacterial strain *Pseudomonas aeruginosa* PAO1 (DSM 22644, ATCC 15692) was cultured and maintained on agar slant cultures in the laboratory using LabM Luria–Bertani (LB) broth solidified with 2% agar. Next, 16 h old (overnight) cell culture was prepared by inoculating 30 mL of LB broth with one loopful of bacterial colony.

The culture was shaken in the dark, at 160 rpm, 30 °C.

### 4.2. Tested Cyclodextrins

As this is the first series of experiments to systematically assess the effect of cyclodextrins on the biofilm formation of *P. aeruginosa*, we aimed to investigate the effect of native cyclodextrins (ACD, BCD, GCD) and to evaluate the effect of some frequently used derivatives.

All these compounds are fine chemicals of CycloLab. In our preliminary experiments on *P. aeruginosa* biofilm formation as well as in our previous research [51] with the *A. fischeri* model organism, the GCD did not show any effect, so we performed this first systematic study with ACD and BCD and their selected derivatives.

**Figure 6:**
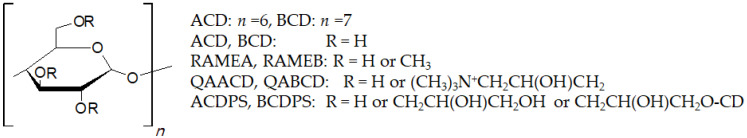
Average molecular formula of the tested cyclodextrins.

The abbreviations (A), the average molecular formula (AMF), the molecular weight (MW), the solubility in water at 25 °C (WS), and the degree of substitution (DS) of the tested CDs are presented in Table 9 and Table 10. The average molecular formula of CDs is illustrated in Figure 6.

The tested CD molecules were suspended in sterile distilled water and after complete dissolution, the stock solutions (or stock suspensions in case of BCD) were sterilized in an autoclave. Then, a dilution series was prepared from 50 mM stock solutions (suspensions) of CDs, covering a 0.4–50 mM concentration range. In the case of polymers, the concentration was related to one-CD-containing units.

### 4.3. Biofilm Formation Assay—Examination of the QQ Effects of Cyclodextrins

The series of experiments aimed to determine the QQ effect of the cyclodextrins was carried out as described by O’Toole with a few modifications [64]. The overnight culture was diluted one-hundred-fold with fresh LB broth. Then, 50 μL of the members of a five-fold cyclodextrin dilution series (0.4 mM, 2 mM, 10 mM, and 50 mM) was added to the wells of a sterile, transparent, 96-well, round-bottomed Sarstedt microtiter plate in five replicates. Distilled water was used as a negative control in the same volume. Thereafter, 150 μL of the diluted overnight culture was added to the wells, except the blank samples for which 150 μL of LB broth was added instead. The microtiter plates were covered and incubated for 6, 24, 48, and 72 h.

To test the effect of temperature on biofilm formation, the microtiter plates were prepared in two replicates to be incubated at 22 and 30 °C, respectively. After the incubation, 150 μL of supernatant from each well was transferred to a new, transparent 96-well microtiter plate. The remaining test medium was removed from cells by turning the plate over and shaking intensely. Then, the plate was gently submerged and washed in a tub of tap water two times in a row without damaging the biofilm inside the wells. After each washing step, the remaining water was shaken and blotted out of the plate. Thereafter, 250 μL of 0.1% crystal violet solution was added to each well of the microtiter plate to stain the biofilm. After 15 min exposure time (at room temperature) the crystal violet solution was removed from the wells and the two-step washing process was repeated. Then, 250 μL of 30% acetic acid solution was pipetted in the wells to solubilize the crystal violet bound by the biofilm. The microtiter plate was incubated at room temperature for 15 min, then 250 μL of the acetic acid solution containing the solubilized crystal violet was transferred to a new, transparent 96-well microtiter plate.

The absorbance was measured with Fluostar Optima BMG Labtech microplate reader at 544 nm wavelength and DIALAB ELx800 ELISA Microplate Reader (Dialab GmbH, Austria) at a wavelength of 630 nm.

### 4.4. Optical Density Assay—Examination of Population Growth

To investigate whether the CDs had cytotoxic effect, the growth of the bacterial population was determined through the measurement of the optical density of the test medium (OD). Additionally, the measurement of the optical density of the transferred supernatant (ODS) was part of the experiments to investigate the proportion of the planktonic cells unable to integrate into the biofilm. The measurements were carried out as described by Molnár et al. [51]. The OD was measured immediately after the incubation period, prior to biofilm formation testing, and ODS was measured with DIALAB ELx800 ELISA Microplate Reader (Dialab GmbH, Austria) at a wavelength of 630 nm immediately after the transfer of the supernatant.

### 4.5. Resazurin Reduction Method (RRM)—Examination of Cell Viability

Metabolic activity was determined as described by Palomino et al. (with a few modifications) [65]. The RRM is based on the reduction of the weakly fluorescent, blue colored resazurin (7-hydroxy-3H-phenoxazin-3-one-10-oxide, Alamar blue) to the highly fluorescent, pink-colored resorufin.

The reaction takes place in the mitochondrial respiratory chain in viable cells, which suggests that the amount of resorufin and the change in the fluorescence intensity is directly proportional to the number of viable bacteria.

Next, 30 µL of 0.5 mM sterile resazurin solution was added to each well containing the 150 μL of supernatant previously removed from the incubated microtiter plate, then it was stored for 15 min in the dark at room temperature. The fluorescence was measured with Fluostar Optima BMG Labtech microtiter plate reader with excitation at 544 nm wavelength and emission at the wavelength of 590 nm.

### 4.6. Statistical Analysis

Repeated measures analysis of variance (RM ANOVA) was performed with TIBCO Statistica™ 13.5 (TIBCO Software, Inc., Palo Alto, CA, USA) software to investigate whether the cyclodextrin concentrations, the exposure time (incubation time), and their interactions affected the biofilm formation of *P. aeruginosa*. Cyclodextrin concentration was considered as a grouping factor and the within-subject factor was the exposure time, which varied within the grouping factor. The Mauchley sphericity test was applied to confirm the criteria. Statistical analyses were performed at the *p* < 0.05 significance level. Tukey’s honestly significant difference test was used for comparison of the effects of the treatments. The significant effects are marked by asterisk (*) in all figures (*p* < 0.05).

Effective Concentration (EC_10,_ EC_20,_ EC_50,_ EC_90_) values causing 10, 20, 50, and 90% inhibition of biofilm formation were determined using OriginPro 2018 software following the concentration–response analysis with Logistic function fitting (y = A2 + (A1 − A2)/(1 + (x/x0)^^p^)).

Pearson Product Moment Correlation Analysis was also performed by TIBCO Statistica™ 13.5 (TIBCO Software, Inc., Palo Alto, CA, USA) to examine the relationship between measured endpoints and CD concentrations. The level of significance was *p* < 0.05. Correlation was considered strong when the correlation coefficient (r) was higher than 0.60 and very strong at r > 0.85

## 5. Conclusions

This work is the first to demonstrate, based on a systematic study, that cyclodextrins can attenuate biofilm formation as a QS-mediated virulence factor of *P. aeruginosa* PAO1.

The potential concentration- and time-dependent quorum quenching ability of different cyclodextrins, which might interfere with the control mechanisms of biofilm formation by *P. aeruginosa*, was clearly shown. The cavity size and the chemical environment of the cavity entrances of cyclodextrins were found to affect bacterial communication via inclusion complex formation of bacterial QS signal molecules. The lower efficiency of cationic cyclodextrins (QAACD, QABCD) was clearly demonstrated compared to the neutral cyclodextrin derivatives. Furthermore, our results also indicated that the effects mediated by random methylated cyclodextrin derivatives was higher compared to native cyclodextrins, which were particularly pronounced at lower concentrations (0.1–2.5 mM).

In terms of methodology, this paper demonstrated the applicability of the microtiter plate biofilm formation assay as a high throughput screening tool for characterizing the influence of additives on biofilm formation not only after the conventional 24 h of incubation, but also extended to 72 h.

The ability of the ACD, RAMEA and RAMEB cyclodextrins to significantly inhibit *Pseudomonas aeruginosa* biofilm formation suggests that cyclodextrin-based solutions may be superior antibiofilm treatments compared to conventional techniques such as antibiotics.

## Figures and Tables

**Table 1:** RMANOVA results over time to evaluate effects of ACD and ACD derivatives on the biofilm formation at 22 °C. Bold numbers indicate significant differences at *p* < 0.05.

Source of Variation	Df ^1^	MS ^2^	F ^3^	*p* ^4^
ACD				
ACD treatment	**4**	**1.14**	**138.57**	**0.000**
Time	**3**	**2.17**	**459.61**	**0.000**
Time × ACD treatment	**12**	**0.16**	**33.41**	**0.000**
RAMEA				
RAMEA treatment	**4**	**1.26**	**175.74**	**0.000**
Time	**3**	**1.19**	**217.22**	**0.000**
Time × RAMEA treatment	**12**	**0.15**	**26.95**	**0.000**
QAACD				
QAACD treatment	**4**	**0.09**	**12.68**	**0.000**
Time	**3**	**3.92**	**500.30**	**0.000**
Time × QAACD treatment	**12**	**0.08**	**10.51**	**0.000**
ACDPS				
ACDPS treatment	**4**	**1.68**	**461.42**	**0.000**
Time	**3**	**1.23**	**444.38**	**0.000**
Time × ACDPS treatment	**12**	**0.26**	**94.76**	**0.000**

^1^ Degree of freedom; ^2^ mean square; ^3^ F-ratio, ^4^ *p*-value.

**Table 2:** RMANOVA results over time to evaluate effects of ACD and ACD derivatives on the biofilm formation at 30 °C. Bold numbers indicate significant differences at *p* < 0.05.

Source of Variation	Df ^1^	MS ^2^	F ^3^	*p* ^4^
ACD				
ACD treatment	**4**	**2.80**	**400.89**	**0.000**
Time	**3**	**0.93**	**59.09**	**0.000**
Time × ACD treatment	**12**	**0.33**	**21.03**	**0.000**
RAMEA				
RAMEA treatment	**4**	**3.74**	**471.10**	**0.000**
Time	**3**	**0.67**	**52.21**	**0.000**
Time × RAMEA treatment	**12**	**0.48**	**37.10**	**0.000**
QAACD				
QAACD treatment	**4**	**0.45**	**8.22**	**0.001**
Time	**3**	**2.97**	**46.82**	**0.000**
Time × QAACD treatment	**12**	**0.08**	**1.31**	**0.244**
ACDPS				
ACDPS treatment	**4**	**0.88**	**172.58**	**0.000**
Time	**3**	**1.22**	**107.87**	**0.000**
Time × ACDPS treatment	**12**	**0.21**	**18.16**	**0.000**

^1^ Degree of freedom; ^2^ mean square; ^3^ F-ratio; ^4^ *p*-value.

**Table 3:** Inhibition of viability of BCD and its derivatives based on resazurin assay.

Degree of Inhibition [%]	BCD	RAMEB	QABCD	BCDPS
12.5 mM concentration	39 ± 2 (72 h)	23 ± 3 (48 h)	31 ± 3 (72 h)	38 ± 1 (48 h), 44 ± 2 (72 h)

**Table 4:** RMANOVA results over time to evaluate the effects of BCD and BCD-derivatives on the biofilm formation at 22 °C. Bold numbers indicate significant differences at *p* < 0.05.

Source of Variation	Df ^1^	MS ^2^	F ^3^	*p* ^4^
BCD				
BCD treatment	**4**	**0.46**	**55.84**	**0.000**
Time	**3**	**3.63**	**395.76**	**0.000**
Time × BCD treatment	**12**	**0.13**	**14.69**	**0.000**
RAMEB				
RAMEB treatment	**4**	**1.71**	**304.23**	**0.000**
Time	**3**	**1.71**	**228.14**	**0.000**
Time × RAMEB treatment	**12**	**0.28**	**37.64**	**0.000**
QABCD				
QABCD treatment	**4**	**0.01**	**5.92**	**0.004**
Time	**3**	**0.86**	**671.35**	**0.000**
Time × QABCD treatment	**12**	**0.01**	**9.18**	**0.000**
BCDPS				
BCDPS treatment	**4**	**0.08**	**93.23**	**0.000**
Time	**3**	**0.30**	**330.56**	**0.000**
Time × BCDPS treatment	**12**	**0.02**	**20.27**	**0.000**

^1^ Degree of freedom; ^2^ mean square; ^3^ F-ratio; ^4^ *p*-value.

**Table 5:** RMANOVA results over time to evaluate the effects of BCD and BCD-derivatives on the biofilm formation at 30 °C. Bold numbers indicate significant differences at *p* < 0.05.

Source of Variation	Df ^1^	MS ^2^	F ^3^	*p* ^4^
BCD				
BCD treatment	**4**	**2.76**	**222.94**	**0.000**
Time	**3**	**2.16**	**134.13**	**0.000**
Time × BCD treatment	**12**	**0.36**	**22.39**	**0.000**
RAMEB				
RAMEB treatment	**4**	**4.29**	**51.89**	**0.000**
Time	**3**	**1.47**	**14.44**	**0.000**
Time × RAMEB treatment	**12**	**0.59**	**5.76**	**0.000**
QABCD				
QABCD treatment	**4**	**0.22**	**45.60**	**0.000**
Time	**3**	**0.25**	**44.79**	**0.000**
Time × QABCD treatment	**12**	**0.17**	**30.35**	**0.000**
BCDPS				
BCDPS treatment	**4**	**0.44**	**15.23**	**0.000**
Time	**3**	**0.76**	**24.48**	**0.000**
Time × BCDPS treatment	**12**	**0.32**	**10.31**	**0.000**

^1^ Degree of freedom; ^2^ mean square; ^3^ F-ratio; ^4^ *p*-value.

**Table 6:** Effective concentrations (EC20) of cyclodextrins causing 20% inhibition of biofilm formation at 22 °C and 30 °C.

Effective Concentration Values—EC_10_ (22 °C) [mM]								
	ACD	RAMEA	QAACD	ACDPS	BCD	RAMEB	QABCD	BCDPS
6 h	0.01	0.29	>12.50	n.d.	3.51	n.d.	n.d.	1.50
24 h	1.49	0.36	n.d.	0.21	1.84	0.45	n.d.	0.02
48 h	3.23	0.60	n.d.	0.77	1.25	0.29	n.d.	n.d.
72 h	0.87	0.58	2.10	0.34	0.79	0.16	n.d.	n.d.
**Effective Concentration Values—EC_10_ (30 °C) [mM]**								
	ACD	RAMEA	QAACD	ACDPS	BCD	RAMEB	QABCD	BCDPS
6 h	5.36	3.04	0.95	0.19	>12.50	3.82	n.d.	0.91
24 h	0.72	0.32	1.37	0.01	0.12	0.06	n.d.	3.56
48 h	0.38	0.05	0.54	0.22	0.16	0.09	n.d.	n.d.
72 h	0.29	0.13	0.70	0.07	0.65	0.29	0.22	0.35

**Table 7:** Effective concentrations (EC_50_) of cyclodextrins causing 50% inhibition of biofilm formation at 22 °C and 30 °C.

	Effective Concentration Values—EC_50_ (22 °C) [mM]							
	ACD	RAMEA	QAACD	ACDPS	BCD	RAMEB	QABCD	BCDPS
6 h	0.03	0.62	>12.50	n.d.	8.50	n.d.	n.d.	>12.50
24 h	3.91	0.84	>12.50	0.21	>12.50	1.20	n.d.	1.11
48 h	7.54	1.29	n.d.	0.77	3.23	0.61	n.d.	>12.50
72 h	1.89	1.25	10.23	0.34	2.39	1.04	n.d.	n.d.
	**Effective Concentration Values—EC_50_ (30 °C) [mM]**							
	ACD	RAMEA	QAACD	ACDPS	BCD	RAMEB	QABCD	BCDPS
6 h	>12.50	>12.50	>12.50	0.36	>12.50	>12.50	n.d.	>12.50
24 h	1.52	0.74	>12.50	>12.5	0.51	0.22	n.d.	>12.50
48 h	0.82	0.09	>12.50	0.48	0.42	0.19	n.d.	>12.50
72 h	0.60	0.29	1.62	0.19	1.30	0.65	0.45	0.78

**Table 8:** Effective concentrations (EC_90_) of cyclodextrins causing 90% inhibition of biofilm formation at 22 °C and 30 °C.

Effective Concentration Values—EC_90_ (22 °C) [mM]								
	ACD	RAMEA	QAACD	ACDPS	BCD	RAMEB	QABCD	BCDPS
6 h	12.49	>12.50	>12.50	n.d.	>12.50	n.d.	n.d.	>12.50
24 h	>12.50	>12.50	>12.50	>12.50	>12.50	>12.50	n.d.	>12.50
48 h	>12.50	>12.50	n.d.	1.50	>12.50	>12.50	n.d.	>12.50
72 h	7.99	3.71	>12.50	>12.50	>12.50	>12.50	n.d.	n.d.
**Effective Concentration Values—EC_90_ (30 °C) [mM]**								
	ACD	RAMEA	QAACD	ACDPS	BCD	RAMEB	QABCD	BCDPS
6 h	>12.50	>12.50	>12.50	>12.50	>12.50	>12.50	n.d.	>12.50
24 h	4.73	2.49	>12.50	>12.50	>12.50	>12.50	n.d.	>12.50
48 h	1.94	0.19	>12.50	>12.50	>12.50	0.65	n.d.	>12.50
72 h	1.31	0.66	>12.50	>12.50	3.11	1.69	5.73	2.51

**Table 9:** Main chemical properties of the tested α-cyclodextrins.

α-Cyclodextrins	A ^1^	AMF ^2^	MW ^3^ [g/mol]	WS ^4^ [g/L]	DS ^5^
Native α-CD	ACD	C_36_H_60_O_30_	972	145	-
Randomly methylated α-CD	RAMEA	C_36_H_60_-nO_30_ · (CH_3_)n	1127	>500	11
Trimethyl-aminopropyl α-CD	QAACD	C_48_H_80_-nO_40_ · (C_6_H_15_ONCl)n	1430	>500	2.5–4
α-CD polymer	ACDPS	-	40,000 *	>500	-

^1^ Abbreviation; ^2^ average molecular formula; n = DS; ^3^ molecular weight; ^4^ water solubility at 25 °C; ^5^ degree of substitution. * The molecular weight of a unit containing one CD molecule is 1390.

**Table 10:** Main chemical properties of the tested β-cyclodextrins.

β-Cyclodextrins	A ^1^	AMF ^2^	MW ^3^ [g/mol]	WS ^4^ [g/L]	DS ^5^
Native β-CD	BCD	C_42_H_70_O_35_	1135	18	-
Randomly methylated β-CD	RAMEB	C_42_H_70_-nO_35_ · (CH_3_)n	1303	>500	12
Trimethyl-aminopropyl β-CD	QABCD	C_42_H_70_-nO_35_ · (C_6_H_15_ONCl)n	1665	>500	3–4
β-CD polymer	BCDPS	-	87,000 *	>500	-

^1^ Abbreviation; ^2^ average molecular formula; n = DS; ^3^ molecular weight; ^4^ water solubility at 25 °C; ^5^ degree of substitution. * The molecular weight of a unit containing one CD molecule is 1620.

## Data Availability

Experimental data are available within this research article and in the related Supplementary Materials.

## Supplementary Materials

The following supporting information can be downloaded at: https://www.mdpi.com/article/10.3390/molecules27113603/s1, S1. Supplementary material—Results of optical density measurement; S2. Supplementary material—Results of correlation analyses.

## Sample Availability

Samples of the compounds are not available from the authors.
