# Supplementary material for: Effect of Cyclodextrins on the Biofilm Formation Capacity of Pseudomonas aeruginosa PAO1
Source: Molecules. 2022 Jun 3;27(11):3603. doi: 10.3390/molecules27113603 (PMC9181962; doi:10.3390/molecules27113603)
Supplement: Supplementary file 1 [file molecules-27-03603-s001.zip › Molecules S1 Supplementary Materials.pdf]

S1. SUPPLEMENTARY MATERIALS

Effect of Cyclodextrins on the Biofilm Formation Capacity of *Pseudomonas aeruginosa* PAO1

Zsófia Berkl [1], Ildikó Fekete-Kertész [1], Kata Buda [1], Emese Vaszita [1], Éva Fenyvesi [2], Lajos Szente [2] and Mónika Molnár [1,*]

[1] Department of Applied Biotechnology and Food Science, Budapest University of Technology and Economics, Budapest, Hungary; berkl.zsofia@vbk.bme.hu (Z.B.); fekete.kertesz.ildiko@vbk.bme.hu (I.F.K.); kata.buda.bk@gmail.com (K.B.); vaszita.emese@vbk.bme.hu (E.V.); molnar.monika@vbk.bme.hu (M.M.)

[2] CycloLab Cyclodextrin R & D Laboratory Ltd., Budapest, Hungary; fenyvesi.e@cyclolab.hu (É.F.); szente@cyclolab.hu (L.S.)

* Correspondence: molnar.monika@vbk.bme.hu

RESULTS OF OPTICAL DENSITY MEASUREMENT

S1. SUPPLEMENTARY MATERIALS - RESULTS OF OPTICAL DENSITY MEASUREMENT

Table S1. Effect of the native α-cyclodextrin on the population growth of *P. aeruginosa*, determined through the measurement of the optical density

T	Tested sample		Optical density [-]			
			6 h	24 h	48 h	72 h
22°C	Control		0.01 ± 0.00	0.22 ± 0.01	0.53 ± 0.01	0.65 ± 0.04
	ACD [mM]	0.1	0.01 ± 0.00	0.26 ± 0.00	0.65 ± 0.02	0.71 ± 0.01
		0.5	0.01 ± 0.00	0.28 ± 0.00	0.64 ± 0.02	0.95 ± 0.01
		2.5	0.01 ± 0.00	0.26 ± 0.01	0.75 ± 0.01	0.91 ± 0.02
		12.5	0.01 ± 0.00	0.24 ± 0.01	0.68 ± 0.01	0.58 ± 0.02
30°C	Control		0.03 ± 0.00	0.68 ± 0.02	0.61 ± 0.02	0.62 ± 0.02
	ACD [mM]	0.1	0.04 ± 0.00	0.69 ± 0.01	0.69 ± 0.03	0.74 ± 0.02
		0.5	0.04 ± 0.00	0.78 ± 0.03	0.75 ± 0.05	0.79 ± 0.03
		2.5	0.04 ± 0.01	0.93 ± 0.02	0.95 ± 0.03	0.87 ± 0.02
		12.5	0.04 ± 0.00	0.73 ± 0.04	0.79 ± 0.04	0.62 ± 0.01

Table S2. Effect of the native α-cyclodextrin on the optical density of the planktonic supernatant of *P. aeruginosa*

T	Tested sample		Optical density of the planktonic supernatant [-]			
			6 h	24 h	48 h	72 h
22°C	Control		0.01 ± 0.00	0.07 ± 0.00	0.36 ± 0.01	0.60 ± 0.02
	ACD [mM]	0.1	0.00 ± 0.00	0.08 ± 0.00	0.31 ± 0.02	0.56 ± 0.02
		0.5	0.00 ± 0.00	0.09 ± 0.00	0.25 ± 0.00	0.72 ± 0.01
		2.5	0.01 ± 0.00	0.10 ± 0.00	0.34 ± 0.01	0.69 ± 0.03
		12.5	0.01 ± 0.00	0.10 ± 0.00	0.39 ± 0.01	0.59 ± 0.01
30°C	Control		0.02 ± 0.00	0.39 ± 0.02	0.47 ± 0.03	0.44 ± 0.02
	ACD [mM]	0.1	0.03 ± 0.00	0.44 ± 0.02	0.59 ± 0.02	0.52 ± 0.02
		0.5	0.03 ± 0.00	0.50 ± 0.02	0.61 ± 0.03	0.56 ± 0.03
		2.5	0.03 ± 0.00	0.61 ± 0.02	0.94 ± 0.01	0.78 ± 0.07
		12.5	0.03 ± 0.00	0.51 ± 0.01	0.78 ± 0.03	0.55 ± 0.04

Table S3. Effect of the randomly methylated α-cyclodextrin on the population growth of *P. aeruginosa*, determined through the measurement of the optical density

T	Tested sample		Optical density [-]			
			6 h	24 h	48 h	72 h
22°C	Control		0.01 ± 0.00	0.22 ± 0.01	0.53 ± 0.01	0.65 ± 0.04
	RAMEA [mM]	0.1	0.01 ± 0.00	0.25 ± 0.01	0.65 ± 0.01	0.74 ± 0.01
		0.5	0.01 ± 0.00	0.20 ± 0.00	0.64 ± 0.01	0.90 ± 0.01
		2.5	0.01 ± 0.00	0.26 ± 0.01	0.68 ± 0.02	0.92 ± 0.01
		12.5	0.01 ± 0.00	0.24 ± 0.00	0.63 ± 0.01	0.79 ± 0.01
30°C	Control		0.03 ± 0.00	0.68 ± 0.02	0.61 ± 0.02	0.62 ± 0.02
	RAMEA [mM]	0.1	0.04 ± 0.01	0.63 ± 0.03	0.73 ± 0.02	0.76 ± 0.03
		0.5	0.04 ± 0.01	0.83 ± 0.01	0.86 ± 0.04	0.64 ± 0.02
		2.5	0.04 ± 0.00	0.81 ± 0.07	0.90 ± 0.04	0.85 ± 0.08
		12.5	0.05 ± 0.00	0.60 ± 0.02	0.78 ± 0.02	0.72 ± 0.03

Table S4. Effect of the randomly methylated α-cyclodextrin on the optical density of the planktonic supernatant of *P. aeruginosa*

T	Tested sample		Optical density of the planktonic supernatant [-]			
			**6 h**	**24 h**	**48 h**	**72 h**
22°C	Control		0.01 ± 0.00	0.07 ± 0.00	0.36 ± 0.01	0.60 ± 0.02
	RAMEA [mM]	0.1	0.01 ± 0.00	0.07 ± 0.00	0.30 ± 0.02	0.63 ± 0.01
		0.5	0.01 ± 0.00	0.07 ± 0.00	0.24 ± 0.01	0.66 ± 0.02
		2.5	0.01 ± 0.00	0.09 ± 0.01	0.35 ± 0.01	0.73 ± 0.01
		12.5	0.01 ± 0.00	0.10 ± 0.00	0.38 ± 0.01	0.68 ± 0.02
30°C	Control		0.02 ± 0.00	0.39 ± 0.02	0.47 ± 0.03	0.44 ± 0.02
	RAMEA [mM]	0.1	0.03 ± 0.00	0.42 ± 0.00	0.58 ± 0.02	0.53 ± 0.50
		0.5	0.03 ± 0.00	0.51 ± 0.03	0.84 ± 0.05	0.50 ± 0.02
		2.5	0.03 ± 0.00	0.51 ± 0.02	0.91 ± 0.03	0.83 ± 0.03
		12.5	0.03 ± 0.00	0.40 ± 0.03	0.73 ± 0.02	0.73 ± 0.06

Table S5. Effect of the trimethyl-aminopropyl α-cyclodextrin on the population growth of *P. aeruginosa*, determined through the measurement of the optical density

T	Tested sample		Optical density [-]			
			**6 h**	**24 h**	**48 h**	**72 h**
22°C	Control		0.08 ± 0.00	0.47 ± 0.02	0.85 ± 0.02	0.85 ± 0.02
	QAACD [mM]	0.1	0.08 ± 0.00	0.48 ± 0.02	0.87 ± 0.02	0.88 ± 0.02
		0.5	0.08 ± 0.00	0.47 ± 0.02	0.93 ± 0.02	0.98 ± 0.02
		2.5	0.07 ± 0.00	0.46 ± 0.01	1.06 ± 0.04	1.15 ± 0.03
		12.5	0.07 ± 0.00	0.52 ± 0.02	1.15 ±0.04	1.24 ± 0.04
30°C	Control		0.31 ± 0.01	0.71 ± 0.02	0.62 ± 0.05	0.67 ± 0.03
	QAACD [mM]	0.1	0.31 ± 0.01	0.72 ± 0.01	0.65 ± 0.02	0.66 ± 0.02
		0.5	0.31 ± 0.00	0.83 ± 0.02	0.72 ± 0.02	0.65 ± 0.01
		2.5	0.31 ± 0.00	1.05 ± 0.02	0.99 ± 0.04	0.75 ± 0.02
		12.5	0.32 ± 0.05	0.98 ± 0.01	1.17 ± 0.03	1.07 ± 0.04

Table S6. Effect of the trimethyl-aminopropyl α-cyclodextrin on the optical density of the planktonic supernatant of *P. aeruginosa*

T	Tested sample		Optical density of the planktonic supernatant [-]			
			**6 h**	**24 h**	**48 h**	**72 h**
22°C	Control		0.05 ± 0.00	0.15 ± 0.01	0.41 ± 0.02	0.45 ± 0.04
	QAACD [mM]	0.1	0.05 ± 0.00	0.18 ± 0.02	0.43 ± 0.02	0.47 ± 0.03
		0.5	0.05 ± 0.00	0.15 ± 0.02	0.48 ± 0.02	0.49 ± 0.03
		2.5	0.05 ± 0.00	0.14 ± 0.02	0.54 ± 0.05	0.68 ± 0.03
		12.5	0.05 ± 0.00	0.26 ± 0.02	0.84 ± 0.03	0.55 ± 0.04
30°C	Control		0.14 ± 0.01	0.53 ± 0.03	0.40 ± 0.05	0.39 ± 0.04
	QAACD [mM]	0.1	0.14 ± 0.01	0.53 ± 0.03	0.36 ± 0.04	0.42 ± 0.03
		0.5	0.13 ± 0.01	0.57 ± 0.05	0.39 ± 0.05	0.35 ± 0.02
		2.5	0.16 ± 0.01	0.70 ± 0.03	0.82 ± 0.04	0.51 ± 0.09
		12.5	0.17 ± 0.0	0.74 ± 0.04	0.93 ± 0.04	0.92 ± 0.06

Table S7. Effect of the α-cyclodextrin polymer on the optical density of the planktonic supernatant of *P. aeruginosa*

T	Tested sample		Optical density [-]			
			6 h	24 h	48 h	72 h
22°C	Control		0.08 ± 0.00	0.47 ± 0.02	0.85 ± 0.02	0.85 ± 0.02
	ACDPS [mM]	0.1	0.08 ± 0.01	0.43 ± 0.01	0.82 ± 0.03	0.84 ± 0.03
		0.5	0.07 ± 0.01	0.44 ± 0.02	0.84 ± 0.03	0.93 ± 0.02
		2.5	0.07 ± 0.00	0.42 ± 0.02	0.76 ± 0.03	0.88 ± 0.02
		12.5	0.09 ± 0.01	0.49 ± 0.02	0.77 ± 0.02	0.88 ± 0.03
30°C	Control		0.31 ± 0.01	0.71 ± 0.02	0.62 ± 0.05	0.67 ± 0.03
	ACDPS [mM]	0.1	0.31 ± 0.01	0.73 ± 0.02	0.67 ± 0.01	0.62 ± 0.02
		0.5	0.30 ± 0.01	0.77 ± 0.03	0.94 ± 0.02	0.68 ± 0.01
		2.5	0.29 ± 0.00	0.75 ± 0.03	0.98 ± 0.03	0.75 ± 0.04
		12.5	0.35 ± 0.02	0.74 ± 0.03	0.93 ± 0.03	0.71 ± 0.04

Table S8. Effect of the α-cyclodextrin polymer on the population growth of *P. aeruginosa,* determined through the measurement of the optical density

T	Tested sample		Optical density of the planktonic supernatant [-]			
			6 h	24 h	48 h	72 h
22°C	Control		0.05 ± 0.00	0.15 ± 0.01	0.41 ± 0.02	0.45 ± 0.04
	ACDPS [mM]	0.1	0.05 ± 0.00	0.11 ± 0.01	0.45 ± 0.01	0.51 ± 0.03
		0.5	0.05 ± 0.00	0.11 ± 0.01	0.45 ± 0.02	0.53 ± 0.01
		2.5	0.05 ± 0.00	0.12 ± 0.00	0.45 ± 0.03	0.49 ± 0.07
		12.5	0.06 ± 0.01	0.22 ± 0.02	0.47 ± 0.02	0.54 ± 0.03
30°C	Control		0.14 ± 0.01	0.53 ± 0.03	0.40 ± 0.05	0.39 ± 0.04
	ACDPS [mM]	0.1	0.14 ± 0.01	0.51 ± 0.02	0.29 ± 0.01	0.36 ± 0.05
		0.5	0.15 ± 0.00	0.44 ± 0.04	0.66 ± 0.03	0.56 ± 0.05
		2.5	0.14 ± 0.01	0.44 ± 0.04	0.66 ± 0.03	0.51 ± 0.04
		12.5	0.16 ± 0.01	0.27 ± 0.02	0.54 ± 0.03	0.55 ± 0.04

Table S9. Effect of the native β-cyclodextrin on the population growth of *P. aeruginosa,* determined through the measurement of the optical density

T	Tested sample		Optical density [-]			
			6 h	24 h	48 h	72 h
22°C	Control		0.01 ± 0.00	0.24 ± 0.00	0.73 ± 0.01	0.85 ± 0.01
	BCD [mM]	0.1	0.01 ± 0.00	0.24 ± 0.01	0.73 ± 0.01	0.89 ± 0.01
		0.5	0.01 ± 0.00	0.25 ± 0.01	0.73 ± 0.01	0.70 ± 0.01
		2.5	0.01 ± 0.00	0.25 ± 0.01	0.73 ± 0.01	0.94 ± 0.02
		12.5	0.01 ± 0.00	0.24 ± 0.00	0.73 ± 0.01	0.88 ± 0.01
30°C	Control		0.06 ± 0.00	0.67 ± 0.02	0.70 ± 0.02	0.64 ± 0.03
	BCD [mM]	0.1	0.06 ± 0.01	0.67 ± 0.01	0.72 ± 0.01	0.65 ± 0.02
		0.5	0.06 ± 0.00	0.70 ± 0.03	0.73 ± 0.02	0.68 ± 0.05
		2.5	0.06 ± 0.01	0.81 ± 0.03	0.94 ± 0.05	0.69 ± 0.03
		12.5	0.08 ± 0.01	0.74 ± 0.01	0.85 ± 0.04	0.72 ± 0.03

Table S10. Effect of the native β-cyclodextrin on the optical density of the planktonic supernatant of *P. aeruginosa*

T	Tested sample		Optical density of the planktonic supernatant [-]			
			6 h	24 h	48 h	72 h
22°C	Control		0.01 ± 0.00	0.09 ± 0.01	0.37 ± 0.03	0.61 ± 0.03
	BCD [mM]	0.1	0.01 ± 0.00	0.08 ± 0.01	0.35 ± 0.01	0.65 ± 0.01
		0.5	0.01 ± 0.00	0.10 ± 0.00	0.33 ± 0.01	0.58 ± 0.02
		2.5	0.01 ± 0.00	0.09 ± 0.01	0.34 ± 0.02	0.65 ± 0.03
		12.5	0.01 ± 0.00	0.08 ± 0.01	0.40 ± 0.03	0.68 ± 0.01
30°C	Control		0.08 ± 0.00	0.44 ± 0.02	0.56 ± 0.03	0.54 ± 0.02
	BCD [mM]	0.1	0.08 ± 0.01	0.44 ± 0.01	0.55 ± 0.04	0.58 ± 0.01
		0.5	0.08 ± 0.01	0.47 ± 0.02	0.62 ± 0.01	0.57 ± 0.03
		2.5	0.08 ± 0.00	0.45 ± 0.03	0.91 ± 0.05	0.77 ± 0.06
		12.5	0.09 ± 0.00	0.44 ± 0.02	0.79 ± 0.03	0.73 ± 0.05

Table S11. Effect of the randomly methylated β-cyclodextrin on the population growth of *P. aeruginosa,* determined through the measurement of the optical density

T	Tested sample		Optical density [-]			
			6 h	24 h	48 h	72 h
22°C	Control		0.01 ± 0.00	0.24 ± 0.00	0.73 ± 0.01	0.85 ± 0.01
	RAMEB [mM]	0.1	0.01 ± 0.00	0.24 ± 0.01	0.72 ± 0.01	0.88 ± 0.01
		0.5	0.01 ± 0.00	0.25 ± 0.01	0.75 ± 0.01	0.96 ± 0.01
		2.5	0.01 ± 0.00	0.24 ± 0.01	0.83 ± 0.01	1.03 ± 0.01
		12.5	0.01 ± 0.00	0.22 ± 0.02	0.89 ± 0.02	1.27 ± 0.03
30°C	Control		0.06 ± 0.00	0.67 ± 0.02	0.70 ± 0.02	0.64 ± 0.03
	RAMEB [mM]	0.1	0.05 ± 0.00	0.68 ± 0.03	0.74 ± 0.01	0.67 ± 0.01
		0.5	0.06 ± 0.00	0.84 ± 0.01	0.72 ± 0.02	0.65 ± 0.03
		2.5	0.06 ± 0.01	0.86 ± 0.02	1.03 ± 0.04	0.93 ± 0.04
		12.5	0.07 ± 0.00	1.06 ± 0.03	1.17 ± 0.03	1.04 ± 0.02

Table S12. Effect of the randomly methylated β-cyclodextrin on the optical density of the planktonic supernatant of *P. aeruginosa*

T	Tested sample		Optical density of the planktonic supernatant [-]			
			6 h	24 h	48 h	72 h
22°C	Control		0.01 ± 0.00	0.09 ± 0.01	0.37 ± 0.03	0.61 ± 0.03
	RAMEB [mM]	0.1	0.01 ± 0.00	0.08 ± 0.01	0.32 ± 0.01	0.67 ± 0.02
		0.5	0.01 ± 0.00	0.09 ± 0.01	0.35 ± 0.02	0.73 ± 0.02
		2.5	0.01 ± 0.00	0.10 ± 0.01	0.38 ± 0.02	0.66 ± 0.04
		12.5	0.01 ± 0.00	0.09 ± 0.00	0.47 ± 0.03	1.16 ± 0.04
30°C	Control		0.08 ± 0.00	0.44 ± 0.02	0.56 ± 0.03	0.54 ± 0.02
	RAMEB [mM]	0.1	0.08 ± 0.00	0.46 ± 0.02	0.50 ± 0.02	0.57 ± 0.03
		0.5	0.08 ± 0.00	0.52 ± 0.01	0.54 ± 0.03	0.55 ± 0.02
		2.5	0.09 ± 0.01	0.54 ± 0.02	0.99 ± 0.06	1.11 ± 0.09
		12.5	0.09 ± 0.00	0.78 ± 0.04	1.13 ± 0.03	1.06 ± 0.08

Table S13. Effect of the trimethyl-aminopropyl β-cyclodextrin on the population growth of *P. aeruginosa*, determined through the measurement of the optical density

T	Tested sample		Optical density [-]			
			6 h	24 h	48 h	72 h
22°C	Control		0.02 ± 0.00	0.40 ± 0.01	0.80 ± 0.02	0.89 ± 0.03
	QABCD [mM]	0.1	0.02 ± 0.00	0.40 ± 0.01	0.81 ± 0.01	0.90 ± 0.03
		0.5	0.01 ± 0.00	0.40 ± 0.02	0.81 ± 0.02	0.95 ± 0.01
		2.5	0.01 ± 0.00	0.39 ± 0.01	0.85 ± 0.02	0.98 ± 0.01
		12.5	0.01 ± 0.00	0.38 ± 0.00	0.82 ± 0.03	0.97 ± 0.03
30°C	Control		0.11 ± 0.02	0.69 ± 0.01	0.59 ± 0.02	0.54 ± 0.03
	QABCD [mM]	0.1	0.13 ± 0.01	0.72 ± 0.01	0.56 ± 0.03	0.55 ± 0.01
		0.5	0.14 ± 0.00	0.75 ± 0.01	0.62 ± 0.01	0.54 ± 0.01
		2.5	0.13 ± 0.00	0.79 ± 0.02	0.92 ± 0.02	0.41 ± 0.01
		12.5	0.13 ± 0.00	0.90 ± 0.05	0.92 ± 0.04	0.96 ± 0.04

Table S14. Effect of the trimethyl-aminopropyl β-cyclodextrin on the optical density of the planktonic supernatant of *P. aeruginosa*

T	Tested sample		Optical density of the planktonic supernatant [-]			
			6 h	24 h	48 h	72 h
22°C	Control		0.01 ± 0.00	0.18 ± 0.02	0.42 ± 0.02	0.97 ± 0.06
	QABCD [mM]	0.1	0.02 ± 0.00	0.15 ± 0.01	0.42 ± 0.01	0.97 ± 0.09
		0.5	0.02 ± 0.00	0.17 ± 0.01	0.42 ± 0.01	1.07 ± 0.05
		2.5	0.01 ± 0.00	0.19 ± 0.01	0.48 ± 0.01	1.10 ± 0.09
		12.5	0.01 ± 0.00	0.18 ± 0.02	0.42 ± 0.03	1.08 ± 0.13
30°C	Control		0.07 ± 0.00	0.38 ± 0.02	0.40 ± 0.03	0.49 ± 0.03
	QABCD [mM]	0.1	0.06 ± 0.00	0.42 ± 0.02	0.44 ± 0.03	0.51 ± 0.07
		0.5	0.07 ± 0.00	0.42 ± 0.01	0.50 ± 0.04	0.48 ± 0.04
		2.5	0.07 ± 0.00	0.33 ± 0.01	0.74 ± 0.01	1.07 ± 0.11
		12.5	0.07 ± 0.00	0.53 ± 0.02	0.52 ± 0.03	1.18 ± 0.17

Table S15. Effect of theβ-cyclodextrin polymer on the population growth of *P. aeruginosa*, determined through the measurement of the optical density

T	Tested sample		Optical density [-]			
			6 h	24 h	48 h	72 h
22°C	Control		0.02 ± 0.00	0.43 ± 0.01	0.87 ± 0.03	0.91 ± 0.01
	BCDPS [mM]	0.1	0.02 ± 0.00	0.43 ± 0.01	0.85 ± 0.01	0.92 ± 0.01
		0.5	0.02 ± 0.00	0.40 ± 0.00	0.85 ± 0.02	0.92 ± 0.01
		2.5	0.02 ± 0.00	0.43 ± 0.01	0.87 ± 0.02	0.93 ± 0.02
		12.5	0.02 ± 0.00	0.44 ± 0.01	0.82 ± 0.02	0.88 ± 0.01
30°C	Control		0.13 ± 0.01	0.69 ± 0.01	0.62 ± 0.04	0.53 ± 0.02
	BCDPS [mM]	0.1	0.16 ± 0.01	0.71 ± 0.03	0.55 ± 0.03	0.56 ± 0.02
		0.5	0.17 ± 0.02	0.81 ± 0.02	0.60 ± 0.03	0.56 ± 0.01
		2.5	0.16 ± 0.01	0.85 ± 0.02	1.00 ± 0.02	0.84 ± 0.02
		12.5	0.25 ± 0.03	0.67 ± 0.02	0.95 ± 0.03	0.78 ± 0.04

Table S16. Effect of the β-cyclodextrin polymer on the optical density of the planktonic supernatant of *P. aeruginosa*

T	Tested sample		Optical density of the planktonic supernatant [-]			
			6 h	24 h	48 h	72 h
22°C	Control		0.02 ± 0.00	0.14 ± 0.01	0.47 ± 0.02	0.60 ± 0.05
	BCDPS [mM]	0.1	0.02 ± 0.00	0.16 ± 0.01	0.50 ± 0.00	0.60 ± 0.07
		0.5	0.02 ± 0.00	0.15 ± 0.02	0.48 ± 0.01	0.65 ± 0.04
		2.5	0.02 ± 0.00	0.17 ± 0.01	0.48 ± 0.01	0.62 ± 0.06
		12.5	0.02 ± 0.00	0.20 ± 0.01	0.50 ± 0.02	0.60 ± 0.05
30°C	Control		0.09 ± 0.01	0.40 ± 0.04	0.36 ± 0.03	0.36 ± 0.03
	BCDPS [mM]	0.1	0.09 ± 0.01	0.41 ± 0.05	0.48 ± 0.02	0.35 ± 0.04
		0.5	0.09 ± 0.01	0.53 ± 0.06	0.32 ± 0.02	0.35 ± 0.01
		2.5	0.10 ± 0.00	0.49 ± 0.02	0.76 ± 0.05	0.67 ± 0.04
		12.5	0.13 ± 0.01	0.44 ± 0.01	0.66 ± 0.03	0.72 ± 0.04
