# Supplementary material for: Effect of Cyclodextrins on the Biofilm Formation Capacity of Pseudomonas aeruginosa PAO1
Source: Molecules. 2022 Jun 3;27(11):3603. doi: 10.3390/molecules27113603 (PMC9181962; doi:10.3390/molecules27113603)
Supplement: Supplementary file 1 [file molecules-27-03603-s001.zip › Molecules S2 Supplementary Materials.pdf]

# S2. SUPPLEMENTARY MATERIALS

# Effect of Cyclodextrins on the Biofilm Formation Capacity of *Pseudomonas aeruginosa* PAO1

**Zsófia Berkl [1], Ildikó Fekete-Kertész [1], Kata Buda [1], Emese Vaszita [1], Éva Fenyvesi [2], Lajos Szente [2] and Mónika Molnár [1,*]**

[1] Department of Applied Biotechnology and Food Science, Budapest University of Technology and Economics, Budapest, Hungary; berkl.zsofia@vbk.bme.hu (Z.B.); fekete.kertesz.ildiko@vbk.bme.hu (I.F.K.); kata.buda.bk@gmail.com (K.B.); vaszita.emese@vbk.bme.hu (E.V.); molnar.monika@vbk.bme.hu (M.M.)

[2] CycloLab Cyclodextrin R & D Laboratory Ltd., Budapest, Hungary; fenyvesi.e@cyclolab.hu (É.F.); szente@cyclolab.hu (L.S.)

* Correspondence: molnar.monika@vbk.bme.hu

## RESULTS OF CORRELATION ANALYSES

Correlation coefficients between tested CD-concentrations and biofilm formation

## S2. SUPPLEMENTARY MATERIALS - RESULTS OF CORRELATION ANALYSES

**Table S1.** Correlation coefficients between tested CD-concentrations and biofilm formation (22 °C)

| *Correlation coefficients (r)* *Marked correlations are significant at p <0.05000* | | | | | | | | |
|---|---|---|---|---|---|---|---|---|
| Contact time | ACD | RAMEA | QAACD | ACDPS | BCD | RAMEB | QABCD | BCDPS |
| *6 h* | -0.683 | -0.517 | -0.241 | -0.260 | -0.752 | 0.424 | 0.674 | -0.648 |
| *24 h* | -0.919 | -0.674 | 0.656 | -0.544 | -0.496 | -0.778 | -0.676 | -0.714 |
| *48 h* | -0.983 | -0.768 | -0.646 | -0.372 | -0.918 | -0.753 | 0.572 | -0.312 |
| *72 h* | -0.899 | -0.683 | -0.897 | -0.572 | -0.729 | -0.709 | 0.260 | -0.453 |

**Table S2.** Correlation coefficients between tested CD-concentrations and biofilm formation (30 °C)

| *Correlation coefficients (r)* *Marked correlations are significant at p <0.05000* | | | | | | | | |
|---|---|---|---|---|---|---|---|---|
| Contact time | ACD | RAMEA | QAACD | ACDPS | BCD | RAMEB | QABCD | BCDPS |
| *6 h* | -0.842 | -0.881 | -0.546 | -0.571 | 0.069 | -0.909 | 0.664 | -0.830 |
| *24 h* | -0.742 | -0.756 | -0.605 | -0.423 | -0.670 | -0.399 | 0.021 | -0.506 |
| *48 h* | -0.660 | -0.688 | -0.575 | -0.560 | -0.638 | -0.571 | 0.273 | -0.076 |
| *72 h* | -0.659 | -0.762 | -0.858 | -0.251 | -0.772 | -0.716 | -0.571 | -0.627 |
